# Supplementary material for: Morphological variation of the early human remains from Quintana Roo, Yucatán Peninsula, Mexico: Contributions to the discussions about the settlement of the Americas
Source: PLoS One. 2020 Jan 29;15(1):e0227444. doi: 10.1371/journal.pone.0227444 (PMC6988924; doi:10.1371/journal.pone.0227444)
Supplement: S1 Text — (DOCX) [file pone.0227444.s002.docx]

**SUPPLEMENTARY INFORMATION 1 – DETAILED CONTEXT OF THE QUINTANA ROO SPECIMENS**

**Naharón**

*Discovery:* The skeletal remains from Naharón were discovered by James Cooke and Gary Walten in the Naranjal cave system. The remains were located 368 meters from the nearest entrance and 22.6 meters deep [1] (Figure 1).

*Conservation*: The skeletal remains were fragile on site, presenting discoloration and weak structure, with noticeable porosity. The bone surface has light erosion near the borders, but the trabecular bone appears well preserved, albeit thinned, with no evidence of mineral absorption. No mineralization of the tissue or deposition of cemented layers are observed. The bones present a few dark brown stained areas on their surface. About 80% of the skeleton is present, some of it fragmented.

*Sex*: The individual is estimated to have been female, based on the small and not protuberant mastoid process, with no rugosity in the jugal canal. The supraorbital margins are slightly rounded and the superciliary arches are barely noticeable. The frontal bone is only slightly convex. The anterior portion of the superior temporal line is clearly marked, forming a sharp edge on top of the orbit and a small notch above the superciliary arches.

*Age at death*: The individual is estimated to have died between 25 and 30 years of age, based on the complete fusion of the long bones’ epiphyses, teeth fully erupted, partial closure of the coronal suture on the temporal sides, and open Lambda and Bregma. The two preserved teeth – left upper second premolar and left superior first molar – show minimal occlusal wear. There is no evidence of degenerative processes, with the exception of osteophytes at the margin of the third cervical vertebral body.

*Pathology*: The second and third cervical vertebrae are fused at the body, as well as at the spinous and transverse processes. The fusion seems to be congenital, although the presence of osteophytes in the body’s margin may be the result of physical stress. The individual has been diagnosed as a probable case of type II Klipell Feil syndrome.

The skull presents light hyperostosis porotica in the area surrounding Lambda, with thickening of the diploë. In this region, there is a slight flattening of the occipital, which may be the result of unintentional cultural flattening of the skull during her childhood. Small cribra orbitalia lesions are present on both orbits. The frontal shows marks of healed fractures and a healed localized infectious process associated to it. Periostitis is also present on the right femur’s neck.

There are possible post-mortem abrasion marks along the frontal and left parietal, running sagittally over the superior temporal line, which may have been the result of the contact with the rocks surrounding the skull.

The cranial surface is well preserved, without noticeable erosion, although there are some taphonomic cracks and flattened regions. Only the cranial vault, very fragmented, and the maxillae are preserved, as well as a few loose teeth. The frontal is practically intact. The right parietal is broken in three large pieces that were reattached to the vault, and the left parietal fragmented into several pieces. Two were reattached to the vault, but at least four pieces could not be refitted to the vault. The occipital has only its posterior portion preserved. The whole base of the skull is missing completely. The right temporal is still present and it articulates with the parietal. The left temporal, although preserved, cannot be reconnected with the vault. The reconstructed vault shows no taphonomic deformation.

The vault has a slight rhomboid shape and has a mesocephalic cranial index (78.31). Its general shape is different from modern and prehistoric Mayan populations, which are predominantly brachicephalic [2]. Naharón has a vault shape similar to Las Palmas and Muknal.

*Body size*: The stature of the individual is estimated to be 140.87 cm, based on the length of the left radius and humerus, and weighted 53.45 kg, based on the maximum diameter of the femur head [3].

**El Pit I**

*Discovery:* The remains of two human individuals were discovered on the debris mount of the Pat Jacinto cenote and are here referred to as the El Pit I and II individuals (Figure 1). The second individual, named El Pit II, is too fragmentary to be included in this study. The El Pit I individual was found at a depth of 40 to 45 m (see [4] for more details regarding the site) and consists of a fragmented cranium, the left and right femora, the left tibia, and the left and right humeri.

*Conservation:* The two skeletal remains were commingled, however we were able to assign several of the most complete postcranial remains to El Pit I, based on their state of preservation.

The cranial vault is the only region of the skull preserved. The frontal bone is marked by a series of postmortem impacts, just above the glabella. The parietals, the posterior portion of the occipital and the temporal bones are well preserved in the specimen. The surface of the bones are slightly affected by chemical dissolution, and there is no evidence of physical erosion or dragging of the material. The bone is brownish-yellow in the region that was in contact with the sediment, and lighter in the parts exposed directly to water.

Isolated bones from the face were also recovered, including portions of the nasals, maxillae and zygomatics. However, they could not be reconnected to the vault. The alveolar portion of the maxillae are severely damaged by chemical erosion, and it is possible that the individual lost teeth antemortem and had the alveoli reabsorbed. Only two teeth were found in situ: right superior second molar and left superior first molar. The right third molar was found isolated.

*Sex:* The nuchal crest is well marked, of grade 4 from the Standards [5]. There are no other diagnostic elements preserved, and as such the individual is considered as a possible male.

*Age at death*: In the absence of other indicators, the degree of closure of the cranial sutures was used to estimate age at death. This suggests that the individual was a young adult, given that they show no sign of closure. If the mandible found is assumed to belong to this individual, it would support this estimate, as it shows the third molars erupted, but occlusal dental wear that is not pronounced.

*Pathology:* There is a small healed lesion in the frontal, above the left superciliary arch. There is also a small exostosis on the superior margin of the left acoustic canal.

The skull is brachicephalic, with a cranial index of 82.85. The nasal index is 47.16, indicating that the individual was mesorhine. In its proportions, the skull is closer to the modern and prehistoric Mayan populations than to the other skulls found in the Quintana Roo.

**Las Palmas**

*Discovery*: This skeleton was discovered by Jim Coke in the Muknal Remote Siphon during a survey dive in 1992 and was reported in 2002 (Figure 1). The bones were located 174m away from Jailhouse cenote, in the lowermost levels of a large chamber at a depth of 24 m. The skeletal remains were covered by a 10 to 60mm thick layer of fine lime mud.

*Conservation*: The Las Palmas skeleton is well preserved, with light brownish-yellow colored bones, and showing a few taphonomic fractures that have not deformed the bones. The bones are very light and fragile. More than 90 percent of its elements are preserved, and the skeleton was mostly articulated when discovered, with only minor disintegration from gravity collapse. The skeleton was lying adjacent to the cave wall, bent to the left, with arms and legs abducted towards the body, suggesting that the body originally was in an upright sitting position. If so, the body must have been somehow fixed, e.g. with a mortuary shroud, or bonded with tissue or ropes, although there is no evidence in the site to support this interpretation. The extreme abduction of the extremities and the upright sitting position suggests an intentional funerary deposition of the individual. The cave also produced abundant charcoal, probably the result of continuous human occupation, and the skeletal remains of a small mammal.

*Sex*: The sex of the individuals is estimated as female, based on several indicators from the complete skeleton. The angle of the greater sciatic notch is wider than 90^o^ and the long bones are gracile, despite well marked muscular insertions. In the skull, the superciliary arches are only slightly marked, the superior margins of the orbit are slightly rounded, the mastoid processes are slightly asymmetrical, with wide and thin with well-developed digastric notches. Although the cranial traits are not decisive, the pelvic morphology is considered a more accurate indicator of sex, justifying the estimation presented.

*Age at death:* Las Palmas’ age was estimated based on traits from the whole skeleton. The pubic symphysis and auricular surface indicate ages between 44-50 and 45-49, according to the methods of Todd (1988, in [6]) and Lovejoy et al. [6, 7], respectively. Most teeth were lost antemortem, and the maxillae show significant alveolar reabsorption, supporting the relatively old age estimate for the individual. All long bones and the basilar suture are completely fused. Osteophytosis is present on the cervical vertebrae. As such, the individual is estimated to have been a middle to old adult (44-50 year) at the time of death.

*Pathology*: The most notorious pathological trait in the individual is the almost complete loss of teeth antemortem. The individual only retained three or four teeth at the time of death. The alveolar bone shows advanced stages of reabsorption, particularly in the anterior portion of the maxillae. Two periapical abscesses are located above each of the superior second molars. There was a possible third abscess also above the left canine.

On the roof of both orbits, very light cribra orbitalia is observed. Around the landmark Lambda, there is a sparse porosity on the occipital and parietals, which could be the result of healed porotic hyperostosis. Over the parietals (especially the right one) there are small shallow depressions where the porosity is concentrated, but it is not clear if this is the result of hyperostosis porotica or an infectious response to other causes.

The skull has an ovoid outline on the superior norm. Based on the cranial indices, the skull classifies as mesocephalic (75.9), closer to the limit with dolicocephalic skulls, hipsicephalic (78.9) and acrocephalic (103.17). The nasal index (57.77) classifies it as leptorhine and the orbital index is microseme (73.8). The Las Palmas skull is moderately elongated, and the facial skeleton is very different from modern and prehistoric Mayan populations. The squamae of both temporals are short and projects anteriorly, connecting to the frontal and separating the parietals from the greater wings from the sphenoid. The individual presents a double right supraorbital foramen and a single closed left supraorbital foramen. Suborbital foramina are also doubled. The left posterior condyle foramen is absent. Mastoid foramina, posterior palatine and accessory palatine foramina are duplicated on both sides as well. The coronal suture is partially closed.

*Body dimension*: Las Palmas was estimated to be 152.06 cm tall and weighted around 58.3 kg.

**Muknal**

*Discovery*: The Muknal skeleton was discovered by one of us (J.A.) in a chamber of the Palmas cave, located approximately 210m away from the entrance of Jailhouse cenote, at 33m of depth (Figure 1). The site at Muknal is characterized by an extensive accumulation of charcoal covering most of the cave floor over an area of 30m long by 2m wide. This sediment layer has 0.1–0.5m thickness and partially covered the skeleton, which was spread out on a horizontally layered limestone rock, about 0.5 m above the cave floor.

*Conservation:* The surface of the bones is slightly altered by the loss of the external tissue layer, due to chemical dissolution and physical abrasion (but without dragging marks). The edges of the bones and fracture borders lack marks of erosion or smoothing. The surface has a brownish-yellow color with the presence of dark stain, possibly from the contact with the charcoal at the bottom of the cave.

Due to postmortem fractures and thinning of the ethmoid and the internal portion of the orbits, only the greater wings of the sphenoid are preserved. The malar presents a postmortem fracture on its inferior portion, with clear edges indicating that the fracture was recent. The maxillae are missing the portion closest to the sphenoid and all the posterior alveolar region, from the first molar on the right side and from the third molar on the left side. The right maxilla is also missing its anterior and superior portions, as well as the contact with the nasal conchae, from the infraorbital border to the inferior border of the maxilla.

Almost all of the cranium base is missing, with the exception of the posterior margin of the foramen magnum. The fractures at the base of the cranium have darkened edges, indicating they are older than the fractures present in the face.

The following superior teeth were lost postmortem: right lateral incisor, right canine, right second premolar, left canine, left first premolar and left first molar. A possible left superior first premolar and some roots of severely worn unidentifiable teeth were recovered.

When the skull was recovered, only the cranial vault and right orbit were preserved. The isolated left malar and the central portion of the maxillae were also recovered and could be reconnected with the vault after its consolidation. However, the face was later separated from the vault due to its fragile state, to avoid future damages.

The mandible is almost complete, with similar coloration as the skull. The right coronoid process is missing and the left one is very deteriorated from chemical dissolution. Both mandibular condyles were fractured. The mandible presents light erosion at the margins of the mandibular gonial angle on both sides.

*Sex*: The superciliary arches are well marked medially, connecting with the glabella to form a robust eminence, grade 4 in the scale from the Standards [5]. The nuchal crest is not pronounced, grade 3 in the scale from Standards. The mastoid process is of medium size, inclined anteriorly, classified as grade 3. The supraorbital margin is thin, slightly rounded and everted (grade 4). The mental eminence was modified by alveolar reabsorption. However, the eminence is pronounced, grades 3-4 from the Standards. Therefore, the individual is considered as a possible male.

*Age at death:* The age at death was estimated based on the dental eruption and closure of the cranial sutures. Despite the loss of the molars antemortem, it is possible to see that the inferior and superior third molars had erupted at the time of death. The few teeth preserved in the mandible show heavy occlusal wear, reaching the phase I of wear from Lovejoy [8], while on the superior teeth the wear is classified as phase H, giving an age-at-death range of 45-55 for the mandible and 40-50 for the maxilla.

The cranial sutures were analyzed following the methods of Lovejoy and Mendl (1984, in [6]), which suggest the age at death as 48.5±10.5 years for the cranial vault sutures and 56±8.5 years for the lateral and inferior cranial vault sutures. Considering the high standard deviations for the cranial suture methods, as well as the degree of wear of the bone tissue, we estimate the age at death of this individual to be between 40 and 45 years.

*Pathology:* The main pathologies observed in the individual are observed in the dentition. The individual shows antemortem tooth loss and alveolar reabsorption of the central incisors, left lateral incisor, left second premolar, left first molar and the three right molars on the superior arcade. Only the right first premolar and left second molar were present, and both show evidence of alveolar retraction and periodontal disease. The teeth show dental wear that reached the pulpar chamber and an occlusal caries on the distal facet of the premolar. There is no evidence of calculus.

Both glenoid cavities present light arthrosis with signs of bone reaction, more pronounced on the left side. The occipital shows a light suprainion lesion with healed bone reaction. Both orbital roofs present severe cribra orbitalia, more pronounced on the right side, with significant bone remodeling associated to them. Around Bregma, there is evidence of healed porotic hiperostosis. In the superior margin of both acoustic canals, but especially on the left side, there are small patches of reactive bone.

The shape of the Muknal skull is ovoid from superior norm, corresponding to a mesocephalic skull (78.48), with narrow nose (40.81; Leptorhine) and low orbits (82.92). In more general terms, the skull is larger than Naharón and Las Palmas. The facial region, however, is more similar to the fragments preserved from Naharón, with narrow nose, while Las Palmas has a wider nose. The low orbits are a common trait to Muknal, Las Palmas and, possibly, Naharón.

*Body proportion*: Estimates of height and weight were not possible, due to the lack of diagnostic skeletal remains.

**References cited**

1. González AHG, Sandoval CR, Terrazas A, Sanvicente MB, Stinnesbeck W, Aviles J, et al. The Arrival of Humans on the Yucatan Peninsula: Evidence from Submerged Caves in the State of Quintana Roo, Mexico. Current Research in the Pleistocene. 2008;25:1-24.

2. Terrazas A, Benavente ME, Stinnesbeck W. Estudio preliminar de tres cráneos tempranos procedentes de cuevas sumergidas de las costa este Quintana Roo. 2o Simposio Internacional el hombre temprano en América: INAH, México; 2006. p. 189-97.

3. Auerbach BM, Ruff CB. Human body mass estimation: A comparison of “morphometric” and “mechanical” methods. Am J Phys Anthropol. 2004;125(4):331-42. doi: 10.1002/ajpa.20032.

4. Hering F, Stinnesbeck W, Folmeister J, Frey E, Stinnesbeck S, Avilés J, et al. The Chan Hol cave near Tulum (Quintana Roo, Mexico): evidence for long-lasting human presence during the early to middle Holocene. Journal of Quaternary Science. 2018;33(4):444-54. doi: 10.1002/jqs.3025.

5. Buikstra JE, Ubelaker D. Standards for data collection from human skeletal remains. . Fayetteville, Arkansas: Arkansas archeological survey research series; 1994.

6. White TD, Black MT, Folkens PA. Human osteology. 3rd ed. San Diego, Calif.: Academic Press; 2012. 662 p.

7. Lovejoy CO, Meindl RS, Pryzbeck TR, Mensforth RP. Chronological metamorphosis of the auricular surface of the ilium: A new method for the determination of adult skeletal age at death. Am J Phys Anthropol. 1985;68(1):15-28. doi: 10.1002/ajpa.1330680103.

8. Lovejoy CO. Dental wear in the Libben population: Its functional pattern and role in the determination of adult skeletal age at death. Am J Phys Anthropol. 1985;68(1):47-56. doi: 10.1002/ajpa.1330680105.
